# Prediction of distant metastasis and specific survival prediction of small intestine cancer patients with metastasis: A population‐based study

**DOI:** 10.1002/cam4.6166

**Published:** 2023-05-31

**Authors:** Jinyi Xu, Zhiyi Yao, Guoliang Liao, Xi OuYang, Shengxun Mao, Jiaqing Cao, Bin Lai

**Affiliations:** ^1^ Nanchang University Nanchang China; ^2^ Department of General Surgery Longnan people's Hospital Longnan China; ^3^ Department of Gastrointestinal Surgery The Second Affiliated Hospital of Nanchang University Nanchang China

**Keywords:** cancer‐specific survival, distant metastases, nomogram, overall survival, small intestine cancer

## Abstract

**Background:**

Small intestine cancer (SIC) is difficult to diagnose early and presents a poor prognosis due to distant metastasis. This study aimed to develop nomograms for diagnosing and assessing the prognosis of SIC with distant metastasis.

**Methods:**

Patients diagnosed with SIC between 2010 and 2015 were included from the Surveillance, Epidemiology and End Results database. Univariate and multifactor analysis determined independent risk factors for distant metastasis and prognostic factors for overall and cancer‐specific survival. We then constructed the corresponding three nomograms and assessed the diagnostic accuracy of the nomograms by net reclassification improvement, receiver operating characteristic curves and calibration curves, assessed the clinical utility by decision curve analysis.

**RESULTS:**

The cohort consisted of 6697 patients, of whom 1299 had distant metastasis at diagnosis. Tstage, Nstage, age, tumor size, grade, and histological type were independent risk factors for distant metastasis. Age, histological type, T stage, N stage, grade, tumor size, whether receiving surgery, number of lymph nodes removed, and the presence of bone or lung metastases were predictors of both overall survival and cancer‐specific survival. The nomograms showed excellent accuracy in predicting distant metastasis and prognosis.

**Conclusion:**

Nomograms were developed and validated for SIC patients with distant metastasis, aiding physicians in making rational and personalized clinical decisions.

## 1 INTRODUCTION

The small intestine comprises 75% of the gastrointestinal tract's length, measuring approximately 5–6 meters, and accounts for over 90% of the gastrointestinal tract's mucosal surface area.^1^ However, small intestine cancer (SIC) is a rare diagnosis, accounting for only 3% of all gastrointestinal malignancies.^2^ Despite this, SIC incidence has increased annually, as demonstrated in Yao et al.'s^3^ study. The incidence of SIC has increased by 130% from 1976 to 2016, accompanied by a relative increase in mortality of 26%, the authors suggest this could signal overdiagnosis in the United States. As many SIC patients may remain asymptomatic until advanced disease detection, coupled with its difficulty in identifying through imaging examination, diagnosis delays often occur, leading to a poor prognosis.^4^ Surveillance, Epidemiology, and End Results data from 2015 to 2019 show that around 56% of SIC patients exhibited locally advanced tumor growth or distant metastases (DM) at initial diagnosis, and the 5‐year relative survival rate for SIC patients with DM was only 42.6%.^5^

Several previous studies have indicated that surgical resection, tumor stage, histologic grade, and DM presence are survival determinants in SIC patients.^6^, ^7^, ^8^ However, these variables mostly serve as single indicators, lacking the ability to accurately predict SIC patient survival rates, let alone those with DM. Currently, there is limited research on the prognosis of SIC patients who develop distant metastases, negatively impacting clinicians' ability to estimate prognosis and implement timely treatment measures based on relevant research results.

Given the small intestine's high resistance to cancer, research on SIC is scarce compared to other gastrointestinal tumors, hindering clinical diagnosis and treatment for SIC patients.^9^ Nonetheless, SIC incidence has been increasing significantly in recent years,^10^ highlighting the urgent need to expedite research on SIC to improve survival rates and reduce its incidence.

Nomograms are a common tool for estimating oncological and medical prognosis, capable of determining individualized risks based on patient and disease characteristics.^11^ Currently, few studies have reported the various risk factors associated with DM development in SIC, and no relevant nomogram has been developed and validated. Previous nomograms for prognostic analysis of SIC have only been built for single histological subtypes, necessitating reliable and well‐performing nomograms for SIC patients necessary to aid clinicians in making optimal clinical decisions by combining the prognosis results derived from nomogram curves. Ultimately, these nomograms can enhance SIC patients' prognosis and quality of life.^12^

The SEER database provides authoritative cancer statistics in the United States, offering a broad path for studying malignant tumors and rare cancers.^13^, ^14^ In this study, we mined clinical characteristic data and modeled DM using SEER cancer registry data for patients diagnosed with SIC from 2010 to 2015. Moreover, we have developed an overall survival (OS) prediction model and a cancer‐specific survival (CSS) prognosis model for SIC patients with DM to provide guidance and support for clinical treatments through large‐sample analysis.

## 2 PATIENTS AND METHODS

### 2.1 Data source and patient selection

The dataset of SIC patients in this study was extracted from the SEER database, which includes data of patients diagnosed with primary SIC from 2010 to 2015.

### 2.2 Inclusion and exclusion criteria

The inclusion criteria we used were as follows: (1) primary tumor site located in the small intestine; (2) detailed clinical statistical variables were available, such as age, sex, race, and surgical information; (3) complete clinicopathological information, including histological type, grading, TNM staging, tumor size; (4) patients diagnosed through autopsy or based on death certificates were excluded from this study.

The variable information extracted included (1) gender; (2) age; (3) race; (4) T stage; (5) N stage; (6) M stage; (7) histological type; (8) histological grade; (9) tumor size; (10) distant metastasis site; (11) surgical information; (12) chemotherapy information; (13) radiotherapy information; (14) number of lymph nodes removed during surgery; (15) survival time; (16) survival status; (17) cause of death, and (18) diagnostic source.

Ultimately, 6697 patients were included in this study, of whom 1299 were diagnosed with distant metastasis. Since the data from SEER are publicly available and de‐identified, this study was exempt from local institutional review board review.

### 2.3 Development and validation of prediction model

Two cohorts were formed in this study: a diagnostic cohort comprising all patients to explore potential risk factors associated with DM and build a predictive nomogram, and a prognostic cohort of 1299 SIC patients with DM (survival time >0) to investigate factors associated with OS and CSS in DM patients and develop new prognostic nomograms.

Both cohorts were randomly divided into a training set (70%) and a validation set (30%), with the training set primarily used to construct the nomogram and the validation set used to validate it.

### 2.4 Statistical analysis

In this study, R software (version 4.2.0) was used for statistical analysis, and *p* < 0.05 on both sides were considered statistically significant.^15^ The SIC patients were randomly divided into training and validation sets using the chi‐squared test to compare variable distribution between the two groups.

For the diagnostic cohort, univariate logistic regression analysis was performed to identify DM‐related risk factors. Variables with *p* < 0.05 in the univariate analysis were then screened for multifactorial logistic regression analysis to identify independent risk factors associated with DM in SIC patients. A new diagnostic nomogram was constructed using the “rms” software package based on the independent risk factors obtained from the multifactorial logistic regression analysis. Calibration and ROC curves were generated for the nomogram and all independent variables in the training and validation sets to assess diagnostic accuracy and sensitivity.^16^, ^17^, ^18^ DCA was used to assess the clinical utility of the model.^19^

For the prognostic cohort, univariate Cox regression analysis was used to identify factors associated with OS and CSS in patients with DM. Significant variables with *p* < 0.05 were included in multifactorial Cox regression analysis to identify independent prognostic factors. Two prognostic nomograms were created to predict OS and CSS in patients with DM based on these independent prognostic factors. Nomograms of OS and CSS were generated at 3, 5, and 7 years, and time‐dependent ROC curves were calculated for nomograms in the training and validation sets. NRI was calculated to demonstrate the superior predictive accuracy of our nomograms compared to single prognostic factors.^20^ Calibration curves and time‐dependent AUCs were evaluated for accuracy and sensitivity. DCA was also plotted to assess clinical utility. Risk score values for OS and CSS were calculated using the hazard function h(t) generated by the Cox model, with the median risk score value as the cutoff point to classify SIC patients with DM into high‐risk and low‐risk groups. Kaplan–Meier (K–M) survival curves and log‐rank tests were conducted to display differences in OS and CSS status between the two groups.

## 3 RESULTS

### 3.1 Baseline characteristics of the study population

Between 2010 and 2015, a total of 6697 SIC patients in the SEER database met the inclusion criteria for this study, with 1299 of them developing DM (Table 1). The patients were randomly divided into a training set (*n* = 4687) and a validation set (*n* = 2010), with a ratio of 7:3. Table 1 displays the demographic and clinicopathological characteristics of patients in both sets, with 33.5% of patients being older than 70 years old. The most common tumor differentiation grade was Grade I, accounting for 56.7% in the training set and 57.0% in the validation set. T3 was the most common T stage, accounting for 38.4% in the training set and 40.0% in the validation set, while N0 was the most common N stage, accounting for 48.7% in the training set and 50.0% in the validation set. Adenoma/adenocarcinoma was the most common histological type, accounting for 87.9% in both sets. The chi‐squared test showed that the bias between the training and validation sets was completely random and not statistically significant, with *p >* 0.05 for each variable in Table 1.

**TABLE 1:** Demographic and clinicopathological characteristics of patients with small intestine cancer.

	All subjects	Training cohort	Validation cohort	χ^2^	*p*
	(*N* = 6697)	(*n* = 4687)	(*n* = 2010)		
Age					
<70	4456 (66.5%)	3124 (66.7%)	1332 (66.3%)	0.077	0.782
≥70	2241 (33.5%)	1563 (33.3%)	678 (33.7%)		
Sex					
Female	3150 (47.0%)	2222 (47.4%)	928 (46.2%)	0.817	0.366
Male	3547 (53.0%)	2465 (52.6%)	1082 (53.8%)		
Race					
White	5368 (80.2%)	3769 (80.4%)	1599 (79.6%)	0.939	0.625
Black	1021 (15.2%)	709 (15.1%)	312 (15.5%)		
Other	308 (4.6%)	209 (4.5%)	99 (4.9%)		
Grade					
Grade I	3804 (56.8%)	2658 (56.7%)	1146 (57.0%)	2.106	0.551
Grade II	1959 (29.3%)	1388 (29.6%)	571 (28.4%)		
Grade III	793 (11.8%)	548 (11.7%)	245 (12.2%)		
Grade IV	141 (2.1%)	93 (2.0%)	48 (2.4%)		
Histology					
8010–8049: epithelial neoplasms, NOS	52 (0.8%)	32 (0.7%)	20 (1.0%)	1.87	0.600
8140–8389: adenomas and adenocarcinomas	5886 (87.9%)	4120 (87.9%)	1766 (87.9%)		
8440–8499: cystic, mucinous and serous neoplasms	235 (3.5%)	165 (3.5%)	70 (3.5%)		
8930–8999: complex mixed and stromal neoplasms	524 (7.8%)	370 (7.9%)	154 (7.7%)		
T Stage					
T1	1032 (15.4%)	706 (15.1%)	326 (16.2%)	4.63	0.201
T2	1190 (17.8%)	842 (18.0%)	348 (17.3%)		
T3	2605 (38.9%)	1801 (38.4%)	804 (40.0%)		
T4	1870 (27.9%)	1338 (28.5%)	532 (26.5%)		
N Stage					
N0	3286 (49.1%)	2282 (48.7%)	1004 (50.0%)	1.895	0.388
N1	3049 (45.5%)	2158 (46.0%)	891 (44.3%)		
N2	362 (5.4%)	247 (5.3%)	115 (5.7%)		
M Stage					
M0	5398 (80.6%)	3804 (81.2%)	1594 (79.3%)	2.986	0.084
M1	1299 (19.4%)	883 (18.8%)	416 (20.7%)		
Tumor size					
<20	2813 (42.0%)	1932 (41.2%)	881 (43.8%)	4.021	0.134
>40	1514 (22.6%)	1078 (23.0%)	436 (21.7%)		
20–40	2370 (35.4%)	1677 (35.8%)	693 (34.5%)		
Surgery					
Yes	6327 (94.5%)	4424 (94.4%)	1903 (94.7%)	0.172	0.679
No	370 (5.5%)	263 (5.6%)	107 (5.3%)		
Chemotherapy					
Yes	1425 (21.3%)	1010 (21.5%)	415 (20.6%)	0.631	0.427
No	5272 (78.7%)	3677 (78.5%)	1595 (79.4%)		
Radiotherapy					
Yes	193 (2.9%)	136 (2.9%)	57 (2.8%)	0.005	0.946
No	6504 (97.1%)	4551 (97.1%)	1953 (97.2%)		

Classification of grade was defined by ICD‐O‐2 (Table 2), while histological type was defined by ICD‐O‐3, and TNM stage was based on the seventh edition of AJCC stage criteria (Table 3).

**TABLE 2:** Grading and differentiation codes defined in ICD‐O‐2.

Grade	
I	Well differentiated; differentiated, NOS
II	Moderately differentiated; moderately differentiated; intermediate differentiation
III	Poorly differentiated; differentiated
IV	Undifferentiated; anaplastic

**TABLE 3:** TNM classification of small intestine cancer (The Seventh Edition AJCC Cancer Staging).

Primary tumor (T)	
Tx	Primary tumor cannot be assessed
T0	No evidence of primary tumor
Tis	Carcinoma in situ
T1a	Tumor invades lamina propria
T1b	Tumor invades submucosa
T2	Tumor invades muscularis propria
T3	Tumor invades through the muscularis propria into the subserosa or into the nonperitonealized perimuscular tissue (mesentery or retroperitoneum) with extension 2 cm or less*
T4	Tumor perforates the visceral peritoneum or directly invades other organs or structures (includes other loops of small intestine, mesentery, or retroperitoneum more than 2 cm, and abdominal wall by way of serosa; for duodenum only, invasion of pancreas)
Regional lymph nodes (*N*)	
Nx	Regional lymph nodes cannot be assessed
N0	No regional lymph node metastasis
N1	1–3 regional lymph node metastases
N2	4 or more regional lymph node metastases
Distant metastasis	
M0	No distant metastasis
M1	Distant metastasis

*Note*: The nonperitonealized perimuscular tissue is, for jejunum and ileum, part of the mesentery and, for duodenum in areas where serosa is lacking, part of the retroperitoneum.

### 3.2 Incidence and risk factors of distant metastasis in small intestinal tumor patients

A total of 1299 SIC patients (19.4%) were identified with DM. Table 4 displays the results of univariate logistic analysis applied to eight potential factors (age, sex, race, histological type, grade, T stage, N stage, and tumor size), which identified six variables associated with DM, including grade, histological type, T stage, N stage, tumor size, and age. Multifactorial logistic analysis showed that older age, larger tumor size, different tumor histologic types, higher N stage, T stage, and grade served as independent risk predictors of DM in SIC patients (Table 4).

**TABLE 4:** Univariate and multifactorial logistic analysis of distant metastasis in patients with small intestine cancer.

	Univariate analysis			Multivariate analysis		
	OR	95% CI	*p*	OR	95% CI	*p*
Age						
<70	Reference					
≥70	0.796	0.695–0.909	0.005*	0.786	0.682–0.903	0.005*
Sex						
Male	Reference					
Female			0.051			
Race						
White	Reference					
Black			0.060			
Other			0.066			
Grade						
I‐II	Reference					
III‐IV	1.754	1.491–2.058	<0.001*	1.253	1.040–1.507	0.045*
Histology						
8010–8049	Reference					
8140–8389	0.234	0.130–0.421	<0.001*	0.327	0.173–0.613	0.003*
8440–8499	0.260	0.133–0.504	<0.001*	0.246	0.122–0.491	<0.001*
8930–8999	0.167	0.089–0.315	<0.001*	0.332	0.169–0.652	0.007*
T						
T1	Reference					
T2	2.587	1.812–3.766	<0.001*	1.893	1.312–2.781	0.005*
T3	5.591	4.083–7.866	<0.001*	3.531	2.523–5.059	<0.001*
T4	10.755	7.858–15.125	<0.001*	6.357	4.518–9.154	<0.001*
N						
N0	Reference					
N1‐N2	2.632	2.308–3.006	<0.001*	1.821	1.569–2.118	<0.001*
Tumor size, mm						
<20	Reference					
20–40	2.619	2.260–3.041	<0.001*	1.482	1.260–1.746	<0.001*
>40	1.991	1.681–2.359	<0.001*	1.125	0.914–1.384	0.349

“*” representative results are statistically significant.

### 3.3 Diagnostic nomogram development and validation

**FIGURE 1:**
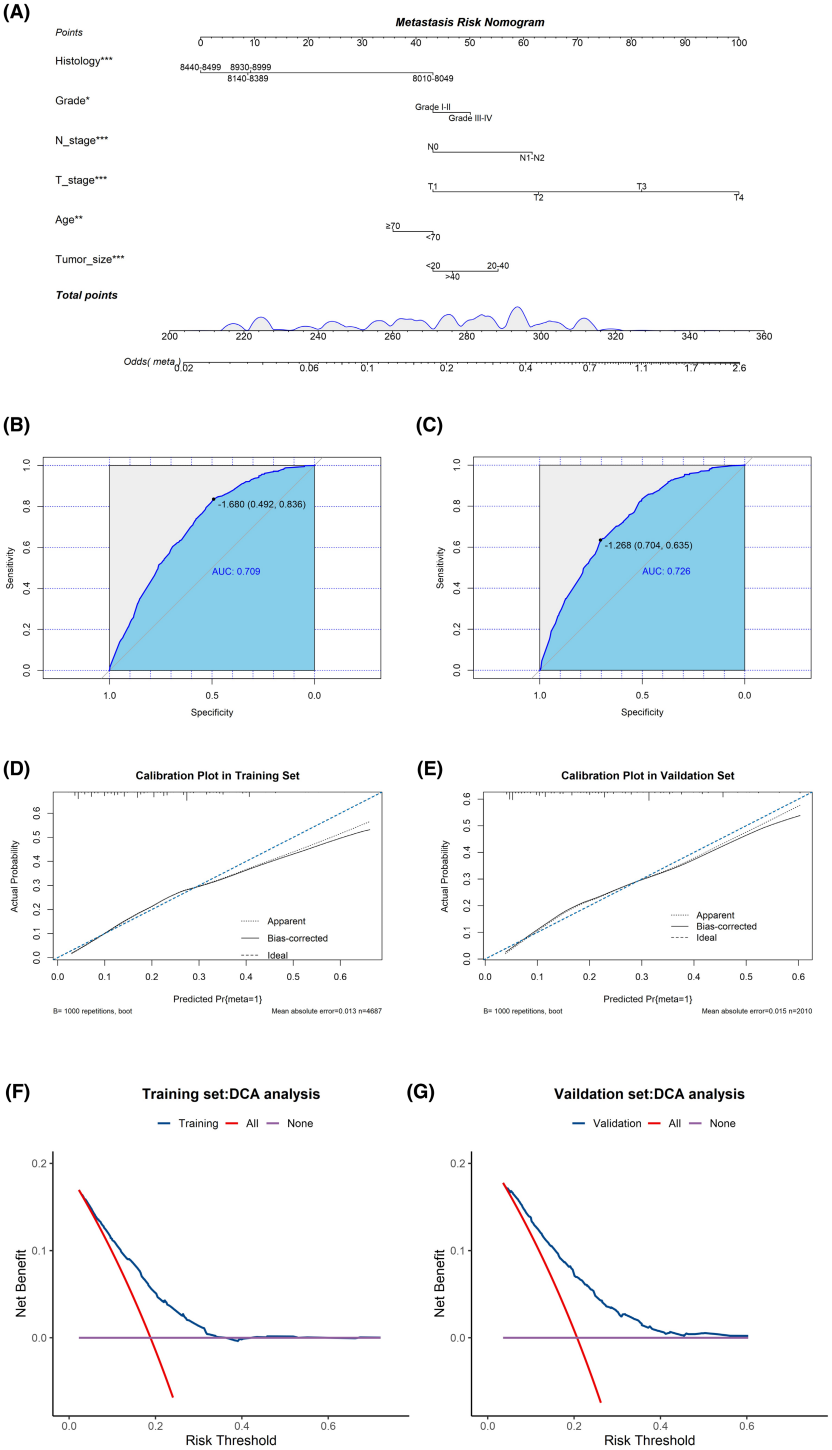
Construction and validation of diagnostic nomogram. Nomogram for DM risk prediction in SIC patients (A). Training set ROC curves (B), calibration curves (D) and DCA (F). Validation set ROC curves (C), calibration curves (E) and DCA (G).

**FIGURE 2:**
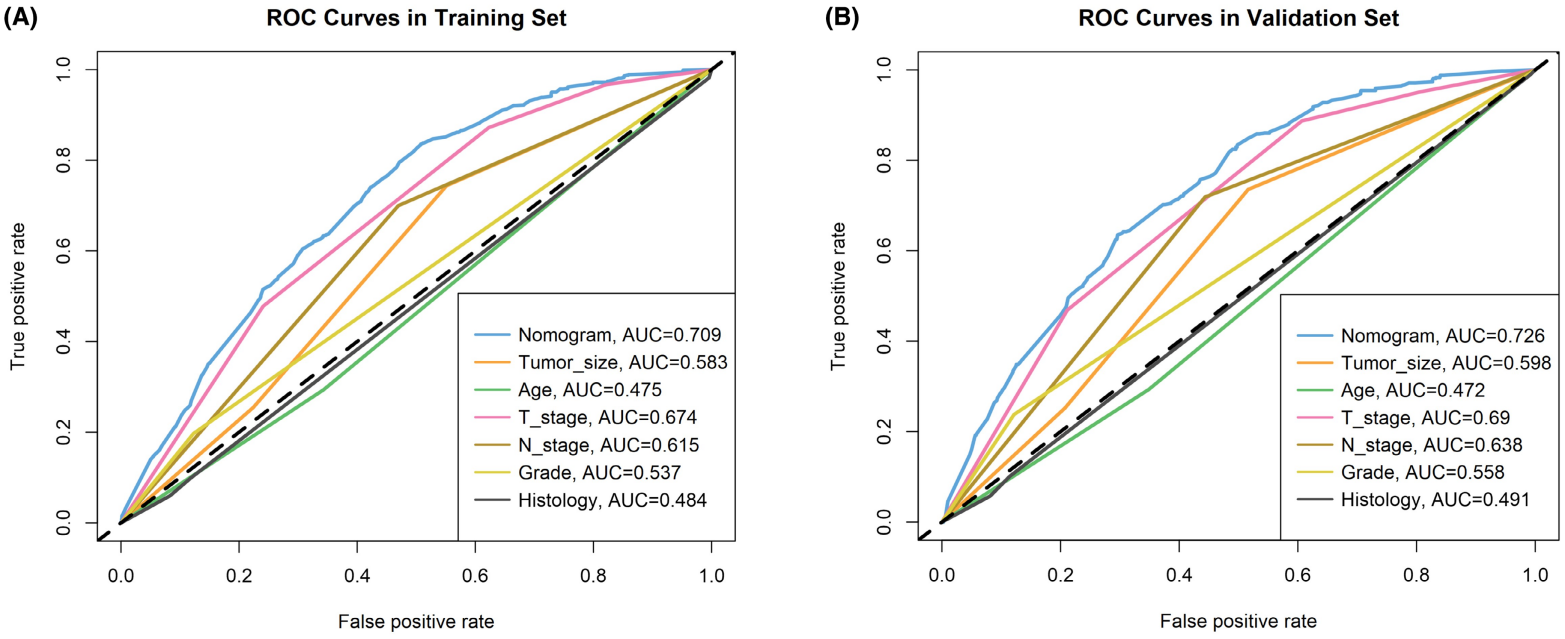
In the training set (A) and validation set (B), nomogram was compared with AUC for the various independent factors (N stage, CS extension, tumor size, and histological type).

A novel nomogram was developed to predict the risk of DM in SIC patients based on six independent predictors (T stage, N stage, age, tumor size, grade, and histological type) (Figure 1A). ROC curves were generated for the training and validation sets, with an AUC of 0.709 and 0.726 in the training and validation sets, respectively (Figure 1B, C). ROC curves for all independent predictors were also constructed, indicating that the newly generated nomogram demonstrated better accuracy than individual factors in both sets (Figure 2). The calibration curves of the nomogram showed excellent agreement between observed and predicted results (Figure 1D, E). DCA curves indicated high clinical net benefit for DM assessment in SIC patients using the diagnostic nomogram developed in this study (Figure 1F, G).

### 3.4 Prognostic factors for small intestinal tumor patients with DM

**FIGURE 3:**
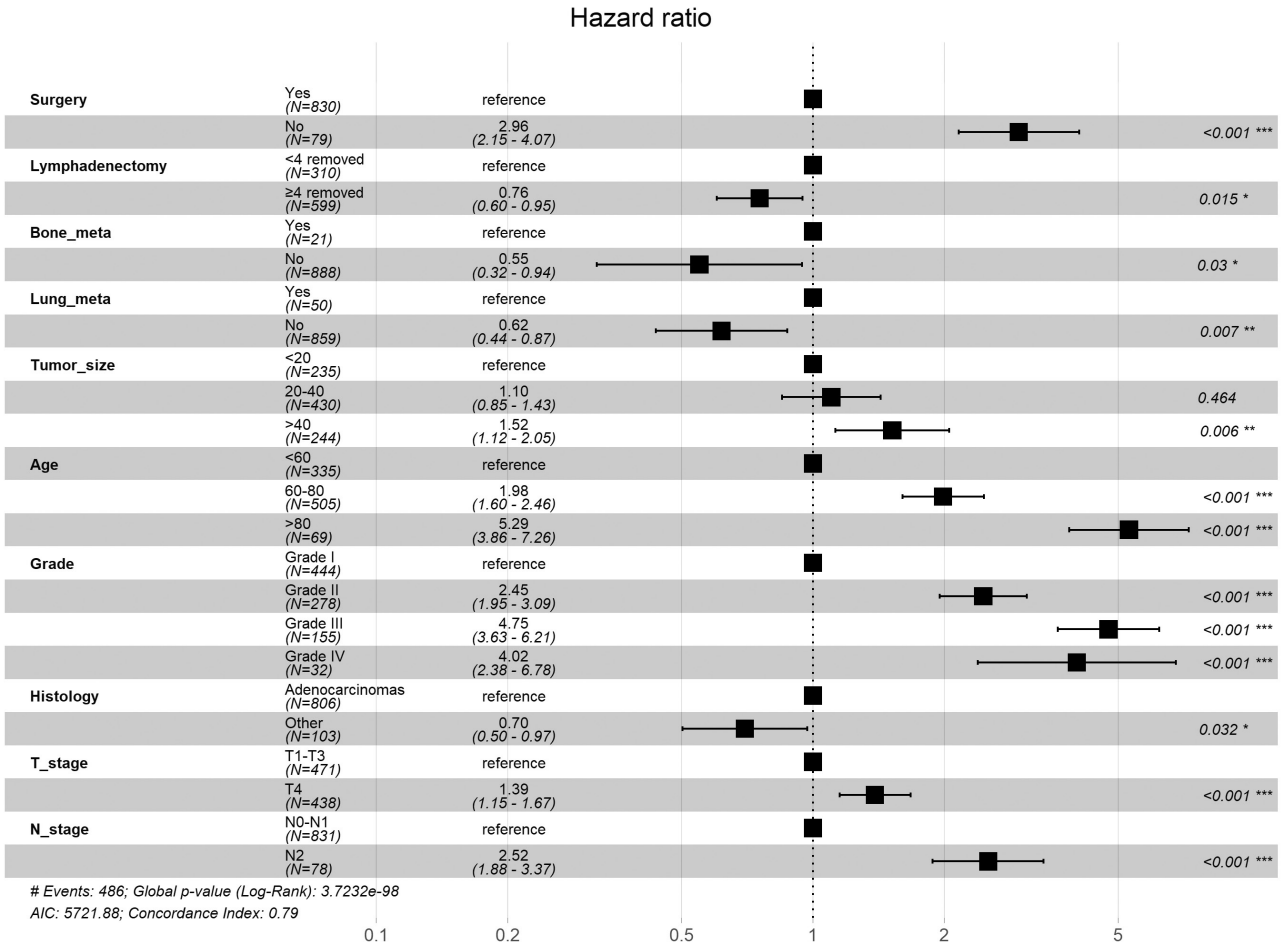
Forest plot of OS in DM patients and p‐value of each factor.

**FIGURE 4:**
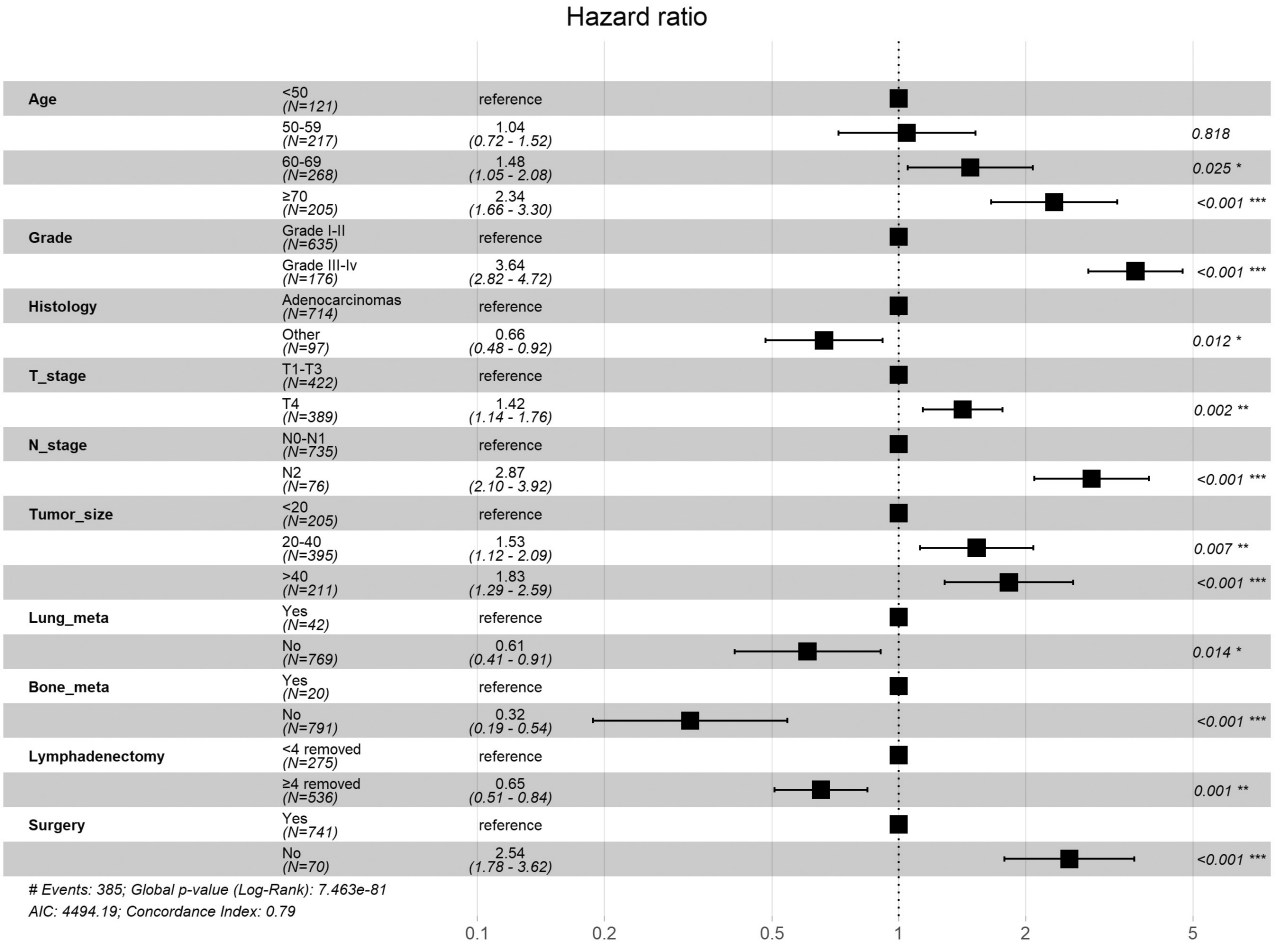
Forest plot of CSS rate in DM patients and *p*‐value of each factor.

The dataset for this study included 1299 SIC patients with DM, among whom the most common age group was 60–80 years (53.8%), the most common tumor differentiation grade was Grade I (49.4%), and the most common N stage was N0‐N1 (91.8%) (Table 5). The chi‐squared test showed that differences in all variables between the training and validation sets were not statistically significant. Univariate and multifactorial Cox regression analyses were used to identify independent prognostic factors for OS and CSS. Forest plots were constructed to illustrate the independent prognostic factors and *p* values for OS (Figure 3) and CSS (Figure 4), respectively. Multifactorial Cox regression models indicated that both OS and CSS were associated with age, histological type, T stage, N stage, grade, tumor size, whether receiving surgery, number of lymph nodes removed, and the presence of bone or lung metastases.

**TABLE 5:** Demographic and clinicopathological characteristics of small intestine cancer patients with DM.

	All subjects	Training cohort	Vaildation cohort	χ^2^	*p*
	(*N* = 1299)	(*n* = 909)	(*n* = 390)		
Age					
<60	501 (38.6%)	335 (36.9%)	166 (42.6%)	4.024	0.134
60–80	699 (53.8%)	505 (55.6%)	194 (49.7%)		
>80	99 (7.6%)	69 (7.6%)	30 (7.7%)		
Sex					
Female	630 (48.5%)	444 (48.8%)	186 (47.7%)	0.103	0.749
Male	669 (51.5%)	465 (51.2%)	204 (52.3%)		
Race					
White	1078 (83.0%)	753 (82.8%)	325 (83.3%)	0.461	0.794
Black	176 (13.5%)	126 (13.9%)	50 (12.8%)		
Other	45 (3.5%)	30 (3.3%)	15 (3.8%)		
Grade					
Grade I	642 (49.4%)	444 (48.8%)	198 (50.8%)	1.588	0.662
Grade II	384 (29.6%)	278 (30.6%)	106 (27.2%)		
Grade III	227 (17.5%)	155 (17.1%)	72 (18.5%)		
Grade IV	46 (3.5%)	32 (3.5%)	14 (3.6%)		
Histology					
Adenocarcinomas	1149 (88.5%)	806 (88.7%)	343 (87.9%)	0.077	0.781
Other	150 (11.5%)	103 (11.3%)	47 (12.1%)		
T stage					
T1‐T3	682 (52.5%)	471 (51.8%)	211 (54.1%)	0.485	0.486
T4	617 (47.5%)	438 (48.2%)	179 (45.9%)		
N stage					
N0‐N1	1192 (91.8%)	831 (91.4%)	361 (92.6%)	0.334	0.563
N2	107 (8.2%)	78 (8.6%)	29 (7.4%)		
Tumor size					
<20	336 (25.9%)	235 (25.9%)	101 (25.9%)	3.728	0.155
20–40	633 (48.7%)	430 (47.3%)	203 (52.1%)		
>40	330 (25.4%)	244 (26.8%)	86 (22.1%)		
Liver meta					
Yes	770 (59.3%)	535 (58.9%)	235 (60.3%)	0.168	0.682
No	529 (40.7%)	374 (41.1%)	155 (39.7%)		
Lung meta					
Yes	66 (5.1%)	50 (5.5%)	16 (4.1%)	0.835	0.361
No	1233 (94.9%)	859 (94.5%)	374 (95.9%)		
Bone meta					
Yes	33 (2.5%)	21 (2.3%)	12 (3.1%)	0.375	0.540
No	1266 (97.5%)	888 (97.7%)	378 (96.9%)		
Brain meta					
Yes	8 (0.6%)	7 (0.8%)	1 (0.3%)	0.487	0.485
No	1291 (99.4%)	902 (99.2%)	389 (99.7%)		
Chemotherapy					
Yes	439 (33.8%)	302 (33.2%)	137 (35.1%)	0.362	0.548
No	860 (66.2%)	607 (66.8%)	253 (64.9%)		
Radiotherapy					
Yes	54 (4.2%)	43 (4.7%)	11 (2.8%)	2.042	0.153
No	1245 (95.8%)	866 (95.3%)	379 (97.2%)		
Lymphadenectomy					
<4 removed	450 (34.6%)	310 (34.1%)	140 (35.9%)	0.313	0.576
≥4 removed	849 (65.4%)	599 (65.9%)	250 (64.1%)		
Surgery					
Yes	1177 (90.6%)	830 (91.3%)	347 (89.0%)	1.485	0.223
No	122 (9.4%)	79 (8.7%)	43 (11.0%)		

### 3.5 Prognostic nomogram development and validation

**FIGURE 5:**
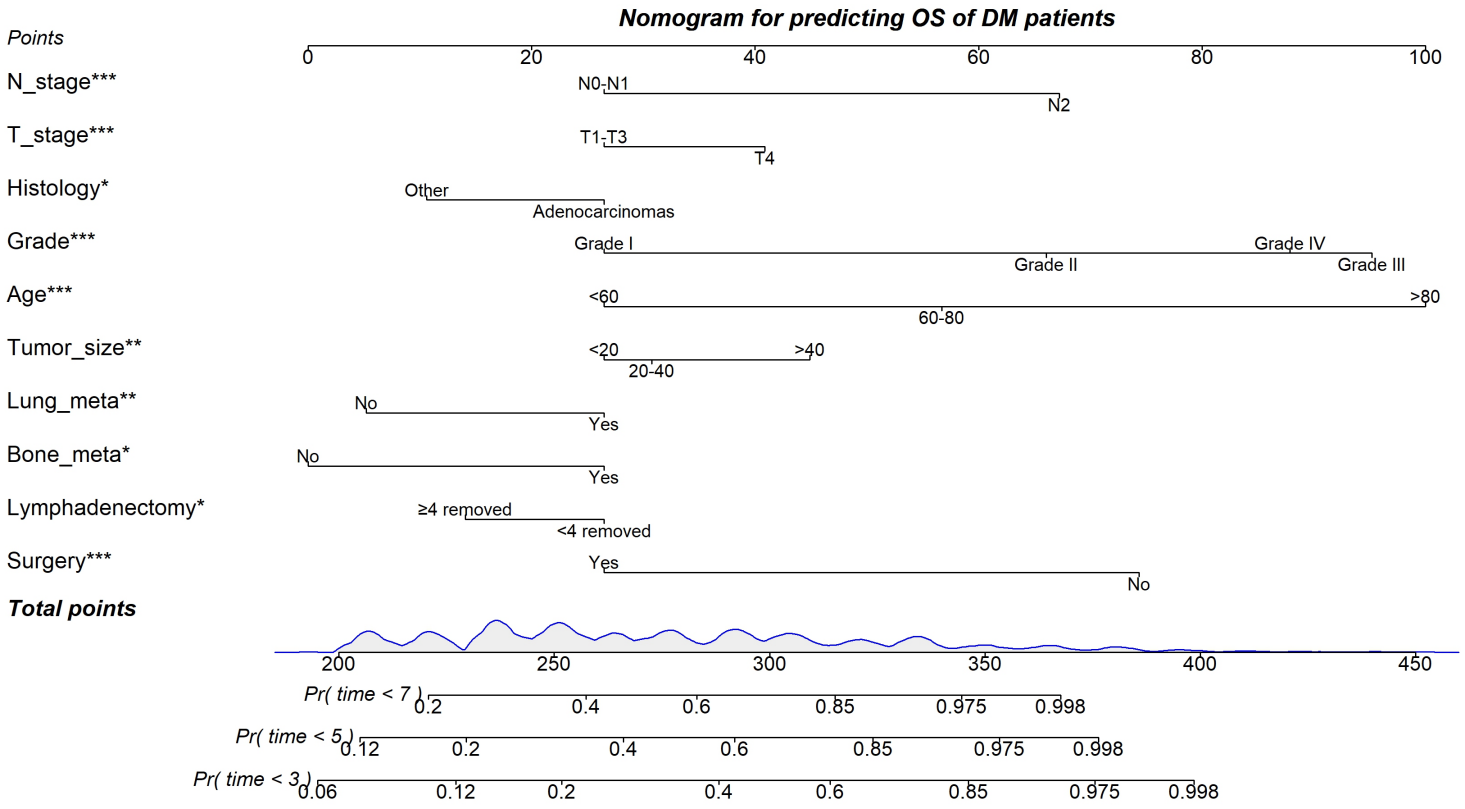
Prognostic nomogram predicting OS at 3, 5 and 7 years in SIC patients.

**FIGURE 6:**
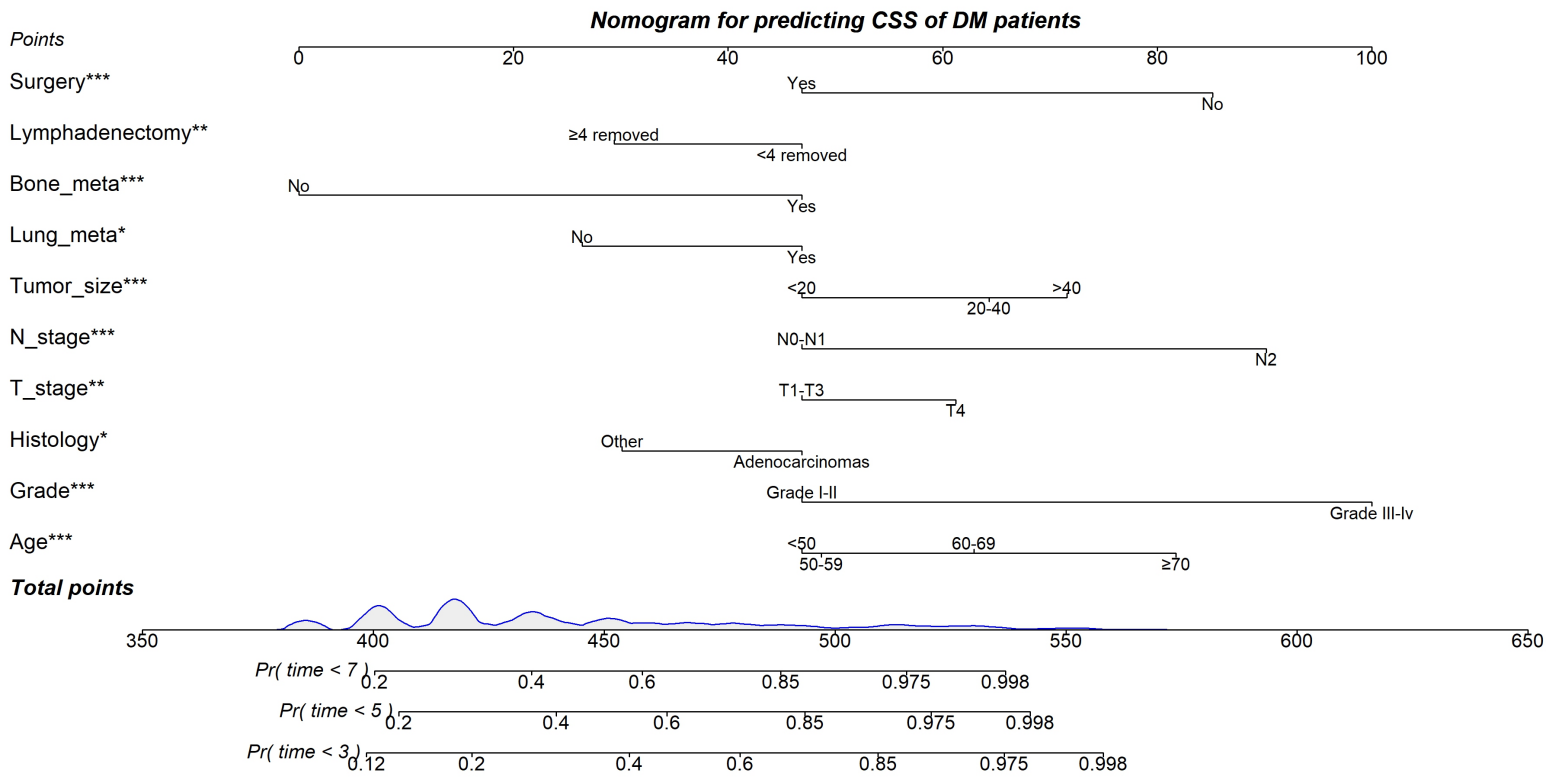
Prognostic nomogram predicting CSS at 3, 5 and 7 years in SIC patients.

**FIGURE 7:**
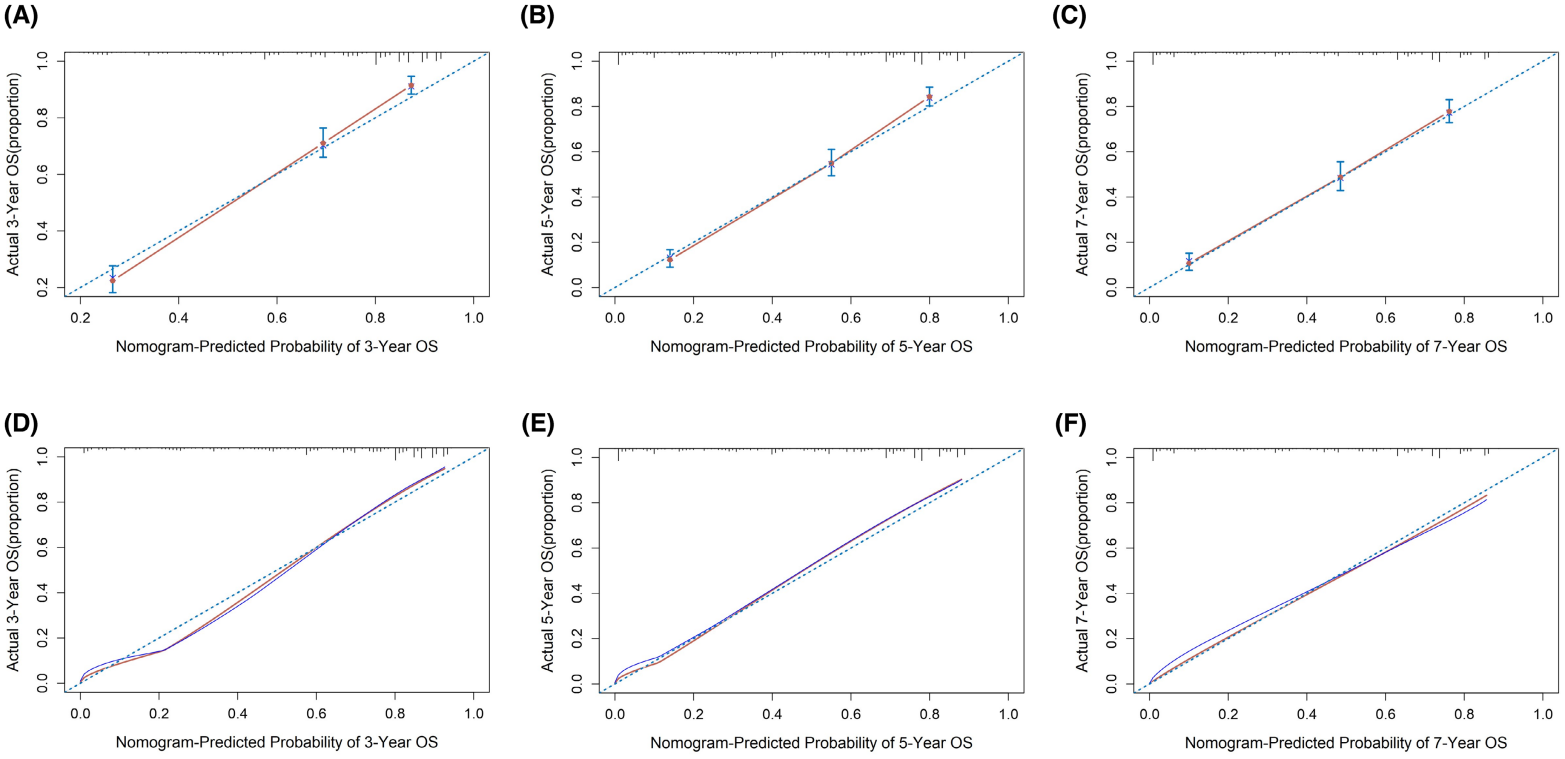
Calibration curves for 3‐, 5‐, and 7‐year OS prediction nomogram in the training set (A, B, and C) and validation set (D, E, and F).

**FIGURE 8:**
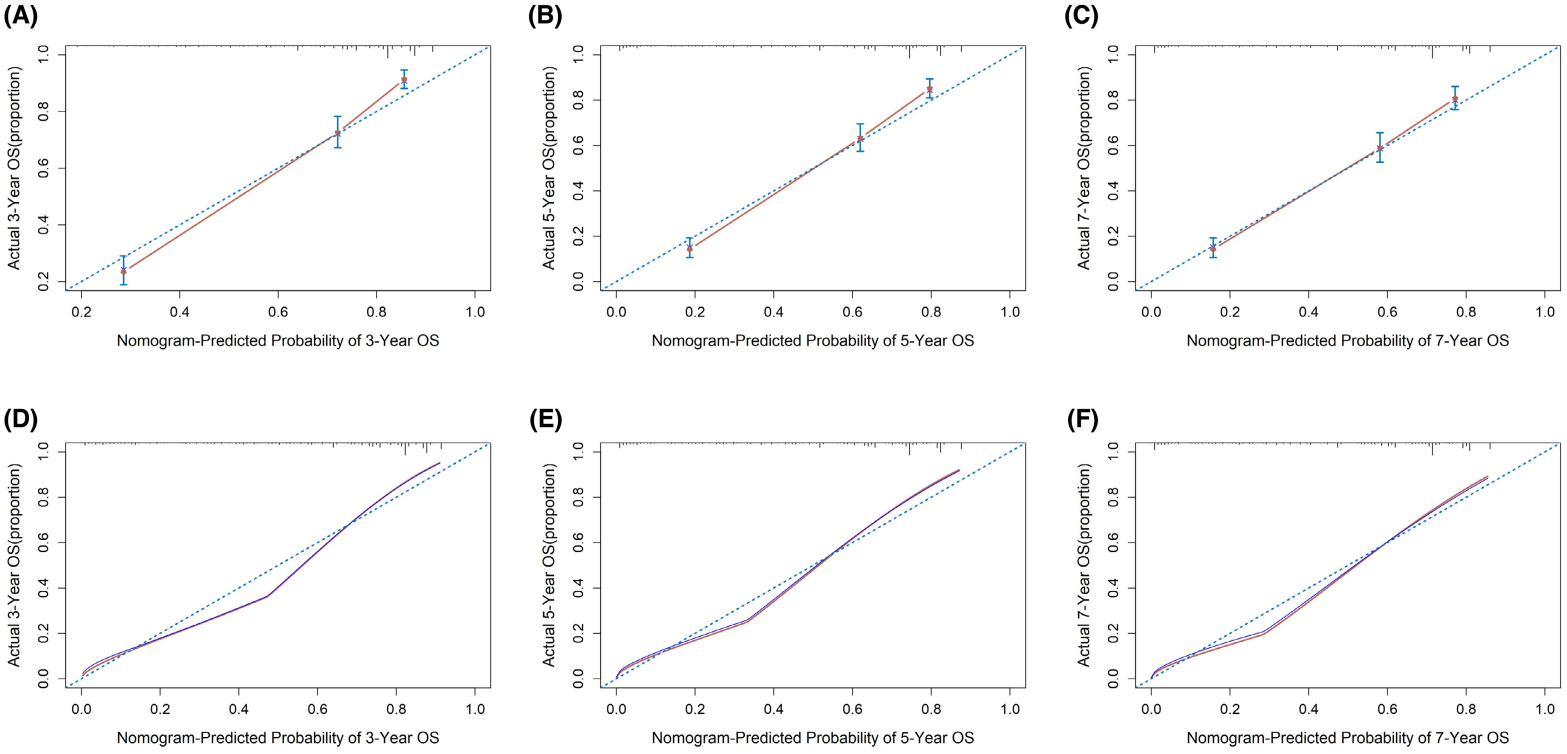
Calibration curves for 3‐, 5‐, and 7‐year CSS prediction nomogram in the training set (A, B, and C) and validation set (D, E, and F).

**FIGURE 9:**
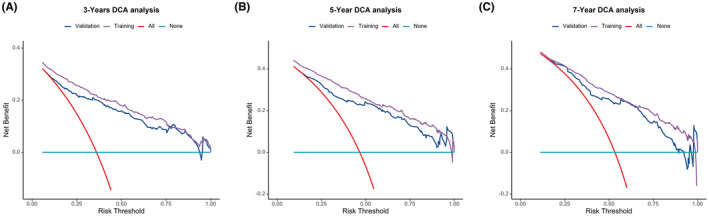
Decision Curve Analysis (DCA) curves for 3‐(A), 5‐(B), and 7‐year(C) OS prediction nomogram in the training and validation sets.

**FIGURE 10:**
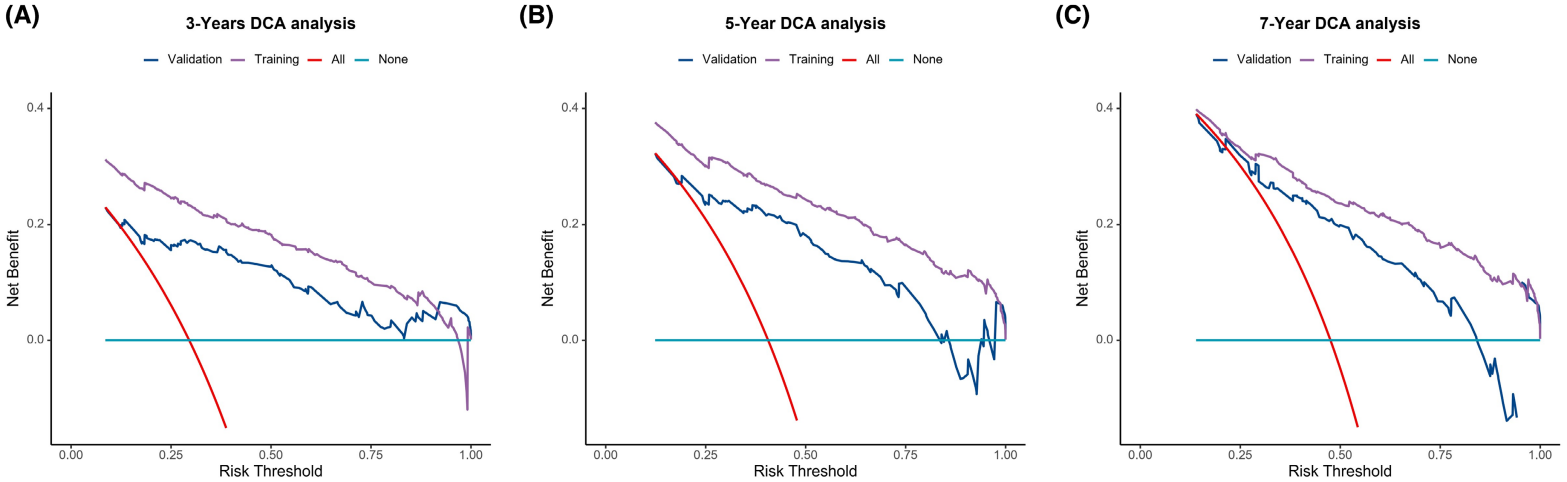
Decision Curve Analysis (DCA) curves for 3‐(A), 5‐(B), and 7‐year(C) CSS prediction nomogram in the training and validation sets.

**FIGURE 11:**
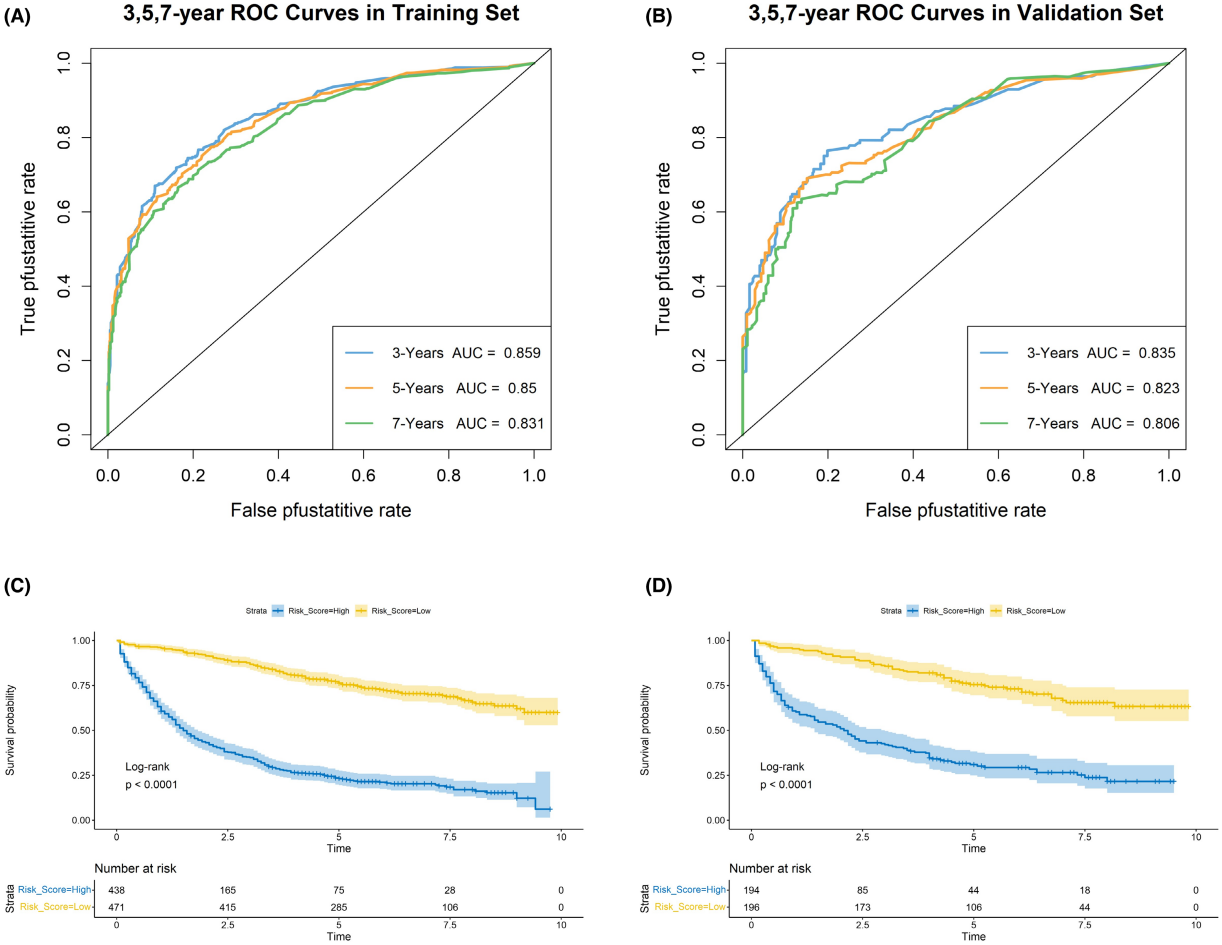
Time‐dependent ROC curve analysis of the OS nomogram for the 3‐, 5‐, and 7‐year in the training set (A) and the validation set (B). The Kaplan–Meier survival curves of the patients in the training set (C) and in the validation set (D).

**FIGURE 12:**
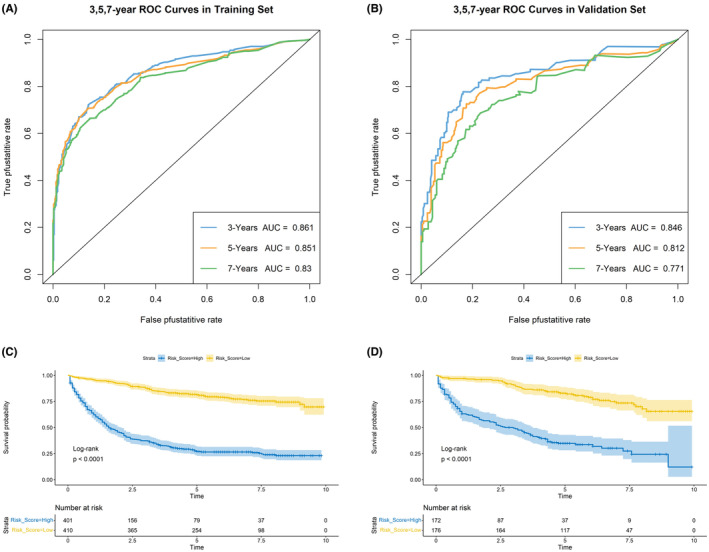
Time‐dependent ROC curve analysis of the CSS nomogram for the 3‐, 5‐, and 7‐year in the training set (A) and the validation set (B). The Kaplan–Meier survival curves of the patients in the training set (C) and in the validation set (D).

Nomograms were constructed to predict OS (Figure 5) and CSS (Figure 6) for SIC patients with DM based on independent prognostic factors derived from multifactorial Cox regression analysis. Calibration curves for the generated nomograms for OS (Figure 7) and CSS (Figure 8) at 3, 5, and 7 years illustrated a higher agreement between the predictions and actual results in both the training and validation sets. DCA curves demonstrated good clinical performance of the predicted nomograms for OS (Figure 9) and CSS (Figure 10). ROC curves for the established patient OS prediction models showed AUCs of 0.859, 0.850, and 0.831 for 3, 5, and 7 years in the training set (Figure 11A), and 0.835, 0.823, and 0.806 in the validation set (Figure 11B), respectively. The AUCs for the patient CSS prediction model ROC curves were 0.861, 0.851, and 0.830 in the training set at 3, 5, and 7 years (Figure 12A), and 0.846, 0.812, and 0.771 in the validation set (Figure 12B), respectively, demonstrating the ability of these nomograms to predict OS and CSS in SIC patients with DM. K–M curves showed that patients in the high‐risk group had significantly lower OS (Figure 11C, D) and CSS (Figure 12C, D) compared to those in the low‐risk group. NRI values were calculated by comparing the nomograms for OS and CSS with nomograms constructed using only their respective single independent prognostic factors, and all NRI >0, indicating significantly better predictive ability of the nomograms in this study compared to individual prognostic factors at 3, 5, and 7 years (Figures S1–S4).

## 4 DISCUSSION

As population‐based screening offers minimal advantages for asymptomatic SIC patients^21^ and a single diagnostic test is often inadequate to establish a conclusive diagnosis,^22^ most SIC patients experience delayed diagnosis, and present with advanced subocclusive crises, leading to a dismal prognosis.^23^, ^24^ Prior research^7^ has demonstrated that DM is a significant prognostic factor in SIC patients, likely due to the fact that primary tumor resection is usually not recommended for DM patients.^25^ However, there remains a considerable research gap concerning the risk factors for DM development in SIC patients and the prognosis of SIC patients with DM. Therefore, the present study aims to create a diagnostic nomogram for DM in SIC patients and a prognostic nomogram for DM patients. By acquiring critical clinical features on the nomogram, we can calculate diagnosis‐related and prognosis‐related scores for patients, allowing us to guide subsequent clinical intervention programs.

In this study, we employed a considerable sample of data featuring meticulous and comprehensive clinical information obtained from the SEER database. Our findings indicate that the likelihood of DM in SIC patients is 19.4%. Furthermore, we have identified six significant predictors of DM in SIC patients, including age, T stage, N stage, tumor size, histological type, and grade. Notably, T stage encodes distinct depth and extent of tumor infiltration. In our research, we found that a high T stage had a positive correlation with the incidence of DM, consistent with previous research indicating that the processes of cell migration and invasion are crucial factors responsible for tumor development and metastasis, contributing to approximately 90% mortality in SIC patients.^26^ Interestingly, patients with tumor diameters exceeding 40 mm exhibited reduced risk of metastasis when compared to those with diameters between 20 and 40 mm. We hypothesize that this might be due to greater heterogeneity of tumor diameters in the “≥40 mm” group of patients. Our study cohort demonstrated that the tumor diameters of SIC patients ranged from 1 mm to 950 mm, with the “≥40 mm” group encompassing a vast range of tumor diameters, resulting in more significant heterogeneity among patients. Moreover, intriguingly, younger patients had increased risk of developing DM than older patients. However, prior studies have reported a prominent relationship between increasing age at diagnosis and reduced risk of distant metastasis across different types of cancer, suggesting that tumor cells in younger patients may be more aggressive.^27^, ^28^, ^29^ Considering the poor prognosis associated with SIC patients with DM, timely detection of DM is vital for patients to undergo appropriate surgical resection, chemotherapy, and radiotherapy.^30^ This nomogram represents the first clinical model specifically designed to predict potential risk of DM in SIC patients, thus filling a critical gap in the field, demonstrating excellent clinical utility through calibration curves, ROC curves, and DCA, which might offer additional reference for the selection of clinical treatment options.

We have further examined the prognostic factors of SIC patients with DM, including OS and CSS. Age, histological type, T stage, N stage, grade, tumor size, whether surgery was performed, the number of lymph nodes removed, and the presence of bone or lung metastases were identified as prognostic factors. We have developed the corresponding prognostic nomogram, indicating that patients with bone or lung metastases may require more aggressive treatments due to significantly lower OS and CSS. Furthermore, patients who received surgical treatment had better outcomes than those who did not, which may provide evidence for clinicians to convince hesitant patients to undergo surgery. Patients who underwent regional lymph node dissection with four or more lymph nodes removed exhibited a better prognosis than those with fewer than four lymph nodes removed, suggesting that expanding the scope of lymph node dissection during surgery should be considered when appropriate.^31^ Although younger SIC patients had a higher risk of distant metastasis than older ones, their prognosis was still better after distant metastasis. Notably, our constructed nomogram for OS indicated a slightly lower risk value for Grade III than for Grade IV, which may be attributed to the small number of Grade IV patients in our dataset, requiring further validation in future studies. It is worth noting that prior studies have reported age and N stage not to be prognostic factors for SIC patients,^6^, ^7^ which differs from our findings. However, other studies suggest that both age and N stage can also serve as prognostic factors for small intestine adenocarcinoma and small intestine carcinoid tumors.^32^, ^33^ These differences may have arisen from the small sample size included in previous studies, inappropriate age grouping of the patients, and the fact that our study focused on SIC patients with DM. Calibration curves, ROC curves, NRI, and DCA demonstrated excellent performance of both prognostic nomograms, with predictive power significantly superior to any independent predictors.

Despite several previous studies on SIC patients creating nomograms, our study significantly improves and expands upon those findings. Our research possesses multiple advantages when compared to existing nomograms for SIC patients.^33^, ^34^, ^35^, ^36^ First, unlike Wang et al^34^ who only studied patients with small intestine adenocarcinoma and Modlin et al^33^ who chose to include patients with small intestine carcinoid tumors, we did not limit ourselves to a single histological subtype and instead selected patients with DM who displayed a poor prognosis and lacked effective treatment, making our study more valuable for clinical guidance. Second, our research included the construction of an innovative predictive model for the development of DM in SIC patients and the prediction of OS and CSS in patients with DM. We also included more clinical characteristics, which made our constructed nomograms more reliable and practical, opening novel avenues for predicting DM that have not been studied thus far. Lastly, our study utilized more common and easily accessible clinical variables, improving the utility and feasibility of the prediction model and providing better AUC values, NRI, and DCA.

However, we must acknowledge that our study still has significant limitations. First, the sample size we examined (*N* = 6697), and further shrinking numbers of SIC patients with DM (*N* = 1229) might result in errors. Second, we constructed the prediction model in the training set and validated it in the validation set, but the nomograms lacked sufficient external data for complete validation, possibly leading to internal bias. Third, relevant information in the SEER database we included was collected only at the time of patient diagnosis, leaving out patients who developed DM at a later stage. Fourth, while race did not act as an independent predictor of the development of DM in SIC patients and the prognosis of patients with DM, our study population was primarily white, rendering our prediction model inapplicable to other populations. In the future, we aim to refine our model by examining data from diverse populations. Furthermore, the predictors in this study only encompassed common clinical variables, as several critical variables such as CEA and CA‐199 were not recorded in the SEER database. Finally, since this is solely a retrospective study, we need to confirm the nomograms designed in this study with relevant prospective studies in the future.

## 5 CONCLUSIONS

This study has developed innovative predictive models for DM in SIC patients, as well as OS and CSS in patients with DM. These three prediction models are of significant clinical importance and can aid clinicians in developing optimal treatment plans for SIC patients, ultimately resulting in more efficient treatment benefits for patients.

## AUTHOR CONTRIBUTIONS

**Jinyi Xu:** Data curation (lead); resources (lead); software (lead); writing – original draft (lead). **Zhiyi Yao:** Data curation (lead); formal analysis (lead); methodology (lead); resources (lead). **Guoliang Liao:** Data curation (equal); formal analysis (equal); funding acquisition (equal); project administration (equal). **Xi OuYang:** Conceptualization (equal); data curation (equal); validation (equal); visualization (equal). **Shengxun Mao:** Data curation (equal); investigation (equal); resources (equal); supervision (equal). **Jiaqing Cao:** Data curation (equal); methodology (equal); project administration (equal); supervision (equal); validation (equal); writing – review and editing (equal). **Bin Lai:** Conceptualization (lead); funding acquisition (lead); project administration (lead); supervision (lead); writing – review and editing (lead).

## ETHICAL APPROVAL

Since the data from SEER are publicly available and de‐identified, this study was exempt from local institutional review board review.

## Supporting information


Figure S1:

## Data Availability

The data that support the findings of this study are available from the corresponding author upon reasonable request.

## References

[1] North JH , Pack MS . Malignant tumors of the small intestine: a review of 144 cases. Am Surg. 2000;66:46‐51. 10651347

[2] Jasti R , Carucci LR . Small Bowel Neoplasms: A Pictorial Review. Radiographics. 2020;40:1020‐1038. doi:10.1148/rg.2020200011 32559148

[3] Yao H , Sokas C , Welch HG . Rising incidence of cancer of the small intestine: overdiagnosis and better diagnosis of low‐lethality disease. Gastroenterology. 2022;162:1749‐1751.e2. doi:10.1053/j.gastro.2022.01.012 35031298

[4] Anzidei M , Napoli A , Zini C , Kirchin MA , Catalano C , Passariello R . Malignant tumours of the small intestine: a review of histopathology, multidetector CT and MRI aspects. Br J Radiol. 2011;84:677‐690. doi:10.1259/bjr/20673379 21586504 PMC3473441

[5] US National Cancer Institute . Surveillance Epidemiology and End Results (SEER) data base . https://seer.cancer.gov

[6] Talamonti MS , Goetz LH , Rao S , Joehl RJ . Primary cancers of the small bowel: analysis of prognostic factors and results of surgical management. Arch Surg. 2002;137:564‐570; discussion 570‐561. doi:10.1001/archsurg.137.5.564 11982470

[7] Brücher BL , Roder JD , Fink U , Stein HJ , Busch R . Prognostic factors in resected primary small bowel tumors. Dig Surg. 1998;15:42‐51. doi:10.1159/000018585 9845562

[8] Taghipour Zahir S , Heidarymeybodi Z , AleSaeidi S . Prognostic Factors and Survival Time in Patients with Small Bowel Tumors: A Retrospective Observational Study. Int J Surg Oncol. 2019;2019:2912361. doi:10.1155/2019/2912361 31186956 PMC6521306

[9] Lamprecht S , Fich A . Small Intestinal Cancer: Why the Rarity? Trends Cancer. 2016;2:395‐397. doi:10.1016/j.trecan.2016.06.006 28741492

[10] Yao H , Sokas C , Welch HG . Rising incidence of cancer of the small intestine: overdiagnosis and better diagnosis of low‐lethality disease. Gastroenterology. 2022;162:1749‐1751.e2. doi:10.1053/j.gastro.2022.01.012 35031298

[11] Balachandran VP , Gonen M , Smith JJ , DeMatteo RP . Nomograms in oncology: more than meets the eye. Lancet Oncol. 2015;16:e173‐e180. doi:10.1016/s1470-2045(14)71116-7 25846097 PMC4465353

[12] Iasonos A , Schrag D , Raj GV , Panageas KS . How to build and interpret a nomogram for cancer prognosis. J Clin Oncol. 2008;26:1364‐1370. doi:10.1200/jco.2007.12.9791 18323559

[13] Yang J , Li Y , Liu Q , et al. Brief introduction of medical database and data mining technology in big data era. J Evid Based Med. 2020;13:57‐69. doi:10.1111/jebm.12373 32086994 PMC7065247

[14] Wu WT , Li YJ , Feng AZ , et al. Data mining in clinical big data: the frequently used databases, steps, and methodological models. Mil Med Res. 2021;8:44. doi:10.1186/s40779-021-00338-z 34380547 PMC8356424

[15] Zhu Y , Fang X , Wang L , Zhang T , Yu D . A predictive nomogram for early death of metastatic gastric cancer: a retrospective study in the SEER database and China. J Cancer. 2020;11:5527‐5535. doi:10.7150/jca.46563 32742500 PMC7391207

[16] Gerds TA , Andersen PK , Kattan MW . Calibration plots for risk prediction models in the presence of competing risks. Stat Med. 2014;33:3191‐3203. doi:10.1002/sim.6152 24668611

[17] Streiner DL , Cairney J . What's under the ROC? An introduction to receiver operating characteristics curves. Can J Psychiatry. 2007;52:121‐128. doi:10.1177/070674370705200210 17375868

[18] Wu J , Zhang H , Li L , et al. A nomogram for predicting overall survival in patients with low‐grade endometrial stromal sarcoma: a population‐based analysis. Cancer Commun (Lond). 2020;40:301‐312. doi:10.1002/cac2.12067 32558385 PMC7365459

[19] Kerr KF , Brown MD , Zhu K , Janes H . Assessing the clinical impact of risk prediction models with decision curves: guidance for correct interpretation and appropriate use. J Clin Oncol. 2016;34:2534‐2540. doi:10.1200/jco.2015.65.5654 27247223 PMC4962736

[20] Leening MJ , Vedder MM , Witteman JC , Pencina MJ , Steyerberg EW . Net reclassification improvement: computation, interpretation, and controversies: a literature review and clinician's guide. Ann Intern Med. 2014;160:122‐131. doi:10.7326/m13-1522 24592497

[21] Kim JS , Park SH , Hansel S , Fletcher JG . Imaging and Screening of Cancer of the Small Bowel. Radiol Clin North Am. 2017;55:1273‐1291. doi:10.1016/j.rcl.2017.06.008 28991566

[22] Rondonotti E , Koulaouzidis A , Georgiou J , Pennazio M . Small bowel tumours: update in diagnosis and management. Curr Opin Gastroenterol. 2018;34:159‐164. doi:10.1097/mog.0000000000000428 29438117

[23] Buckley JA , Fishman EK . CT evaluation of small bowel neoplasms: spectrum of disease. Radiographics. 1998;18:379‐392. doi:10.1148/radiographics.18.2.9536485 9536485

[24] Maglinte DD , O'Connor K , Bessette J , Chernish SM , Kelvin FM . The role of the physician in the late diagnosis of primary malignant tumors of the small intestine. Am J Gastroenterol. 1991;86:304‐308. 1998312

[25] Puccini A , Battaglin F , Lenz HJ . Management of advanced small bowel cancer. Curr Treat Options Oncol. 2018;19:69. doi:10.1007/s11864-018-0592-3 30397729 PMC7489287

[26] Peng HY , Yu QF , Shen W , et al. Knockdown of ELMO3 suppresses growth, invasion and metastasis of colorectal cancer. Int J Mol Sci. 2016;17:2119. doi:10.3390/ijms17122119 27999268 PMC5187919

[27] Balch CM , Thompson JF , Gershenwald JE , et al. Age as a predictor of sentinel node metastasis among patients with localized melanoma: an inverse correlation of melanoma mortality and incidence of sentinel node metastasis among young and old patients. Ann Surg Oncol. 2014;21:1075‐1081. doi:10.1245/s10434-013-3464-x 24531700 PMC4121329

[28] Panda S , Mohanty N , Panda S , et al. Are survival outcomes different for young and old patients with oral and oropharyngeal squamous cell carcinoma?A Systematic Review and Meta‐Analysis. Cancers (Basel). 2022;14:1886. doi:10.3390/cancers14081886 35454794 PMC9029651

[29] Purushotham A et al. Age at diagnosis and distant metastasis in breast cancer‐‐a surprising inverse relationship. Eur J Cancer. 2014;50:1697‐1705. doi:10.1016/j.ejca.2014.04.002 24768572

[30] Zhou Z , Ge H , Li Y , Wang D , Gungor C . Survival effects of primary and metastatic surgical treatment in metastatic small intestinal tumors: a propensity score‐matching study. PLoS One. 2022;17:e0270608. doi:10.1371/journal.pone.0270608 35749551 PMC9231803

[31] Pasquer A , Walter T , Hervieu V , et al. Surgical management of small bowel neuroendocrine tumors: specific requirements and their impact on staging and prognosis. Ann Surg Oncol. 2015;22(Suppl 3):S742‐S749. doi:10.1245/s10434-015-4620-2 26014153

[32] Zheng Z , Zhou X , Zhang J , et al. Nomograms predict survival of patients with small bowel adenocarcinoma: a SEER‐based study. Int J Clin Oncol. 2021;26:387‐398. doi:10.1007/s10147-020-01813-8 33113018

[33] Modlin IM , Gustafsson BI , Pavel M , Svejda B , Lawrence B , Kidd M . A nomogram to assess small‐intestinal neuroendocrine tumor ('carcinoid') survival. Neuroendocrinology. 2010;92:143‐157. doi:10.1159/000319784 20733279

[34] Wang D , Li C , Li Y , et al. Specific survival nomograms based on SEER database for small intestine adenocarcinoma. Ann Palliat Med. 2021;10:7440‐7457. doi:10.21037/apm-21-600 34263641

[35] Wang N , Yang J , Lyu J , et al. A convenient clinical nomogram for predicting the cancer‐specific survival of individual patients with small‐intestine adenocarcinoma. BMC Cancer. 2020;20:505. doi:10.1186/s12885-020-06971-6 32487033 PMC7268250

[36] Gu Y , Deng H , Wang D , Li Y . Metastasis pattern and survival analysis in primary small bowel adenocarcinoma: a SEER‐Based Study. Front Surg. 2021;8:759162. doi:10.3389/fsurg.2021.759162 34950695 PMC8691381

