# Supplementary material for: Prediction of distant metastasis and specific survival prediction of small intestine cancer patients with metastasis: A population‐based study
Source: Cancer Med. 2023 May 31;12(14):15037–53. doi: 10.1002/cam4.6166 (PMC10417179; doi:10.1002/cam4.6166)
Supplement: Supplementary file 1 — Figure S1: [file CAM4-12-15037-s001.docx]

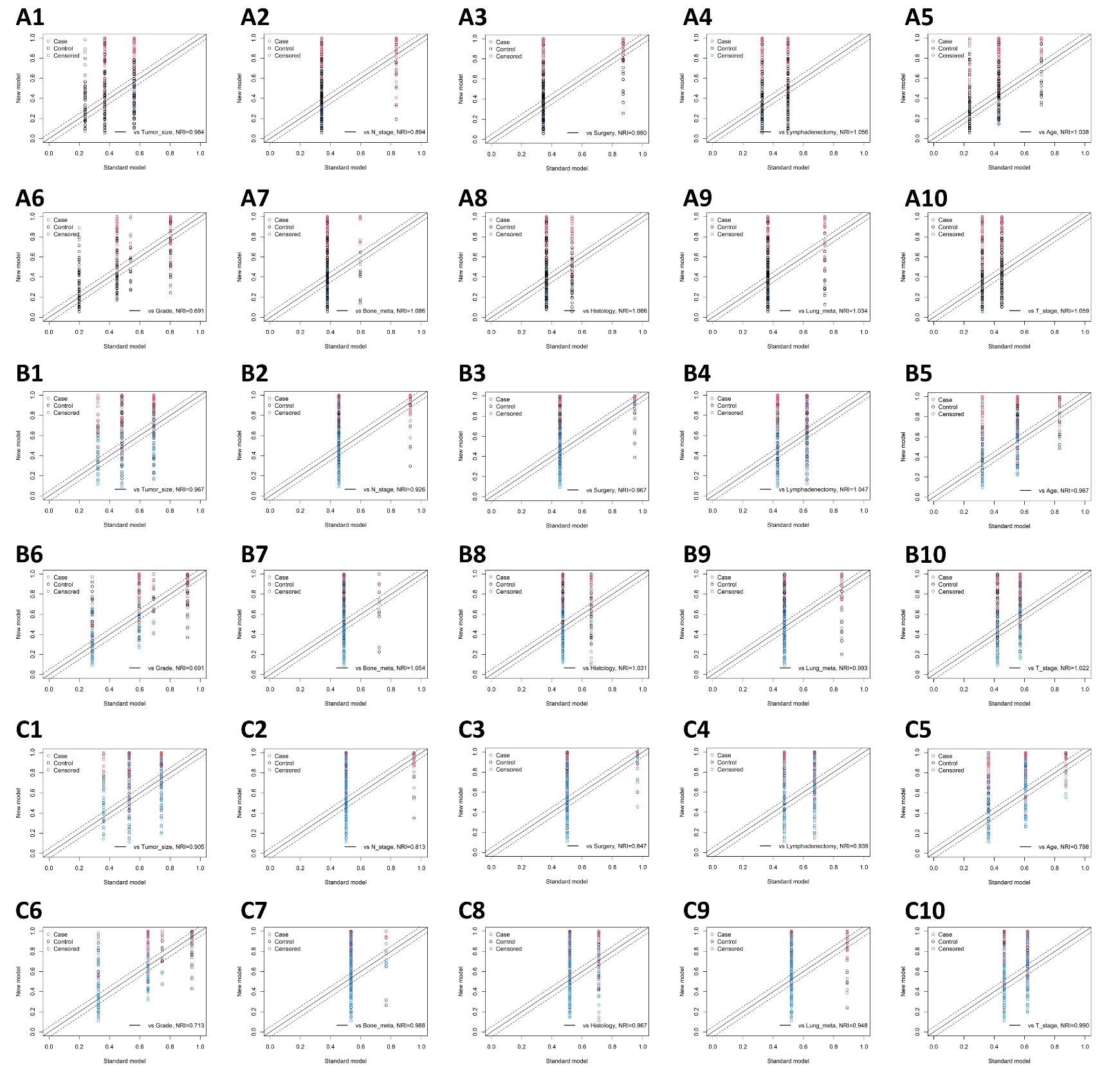


Supplementary Figure 1: Comparison of OS nomogram with NRI for all independent factors for 3 years (A1-10), 5 years (B1-10) and 7 years (C1-10) in the training sets.


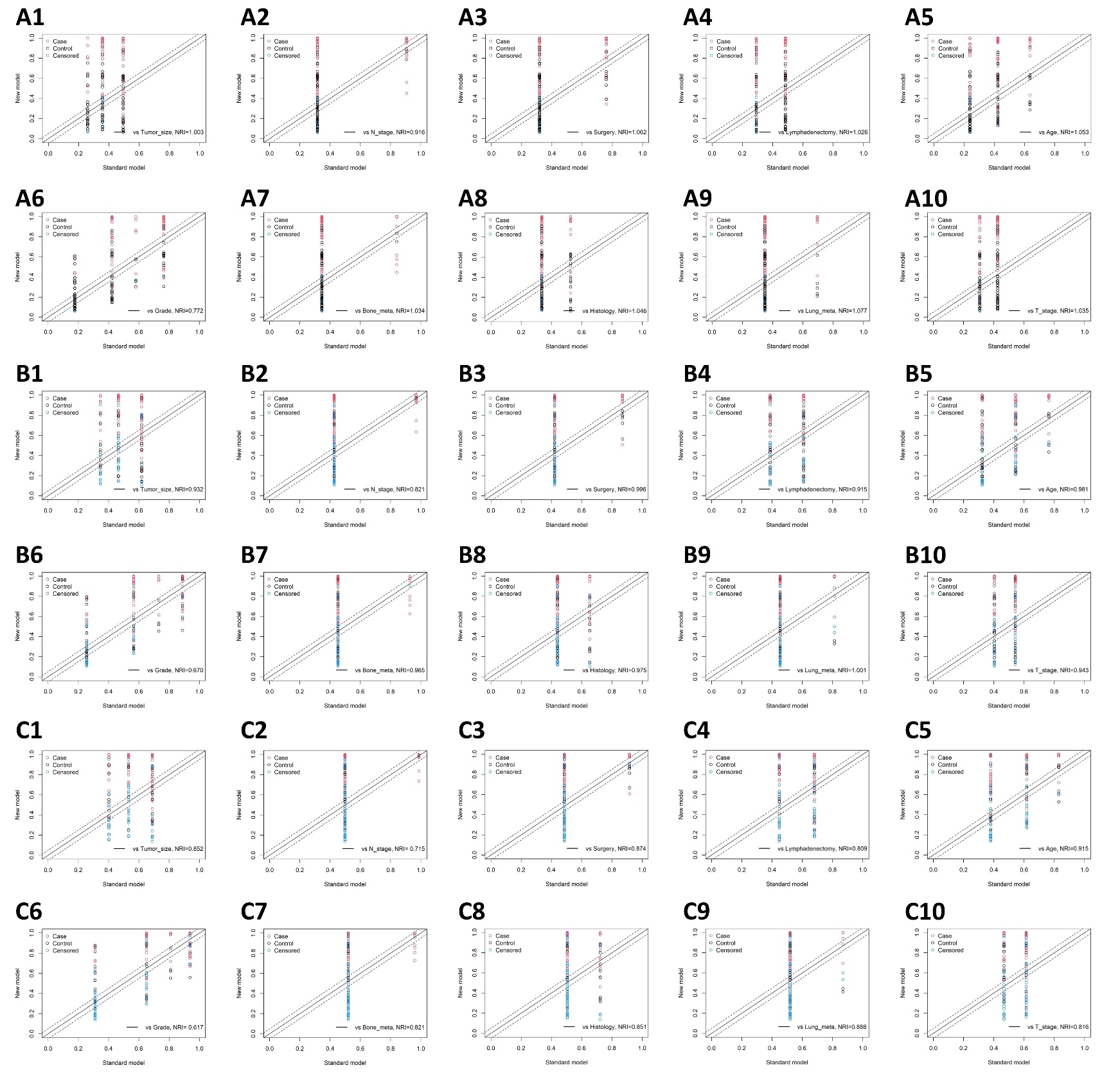


Supplementary Figure 2: Comparison of OS nomogram with NRI for all independent factors for 3 years (A1-10), 5 years (B1-10) and 7 years (C1-10) in the validation sets.


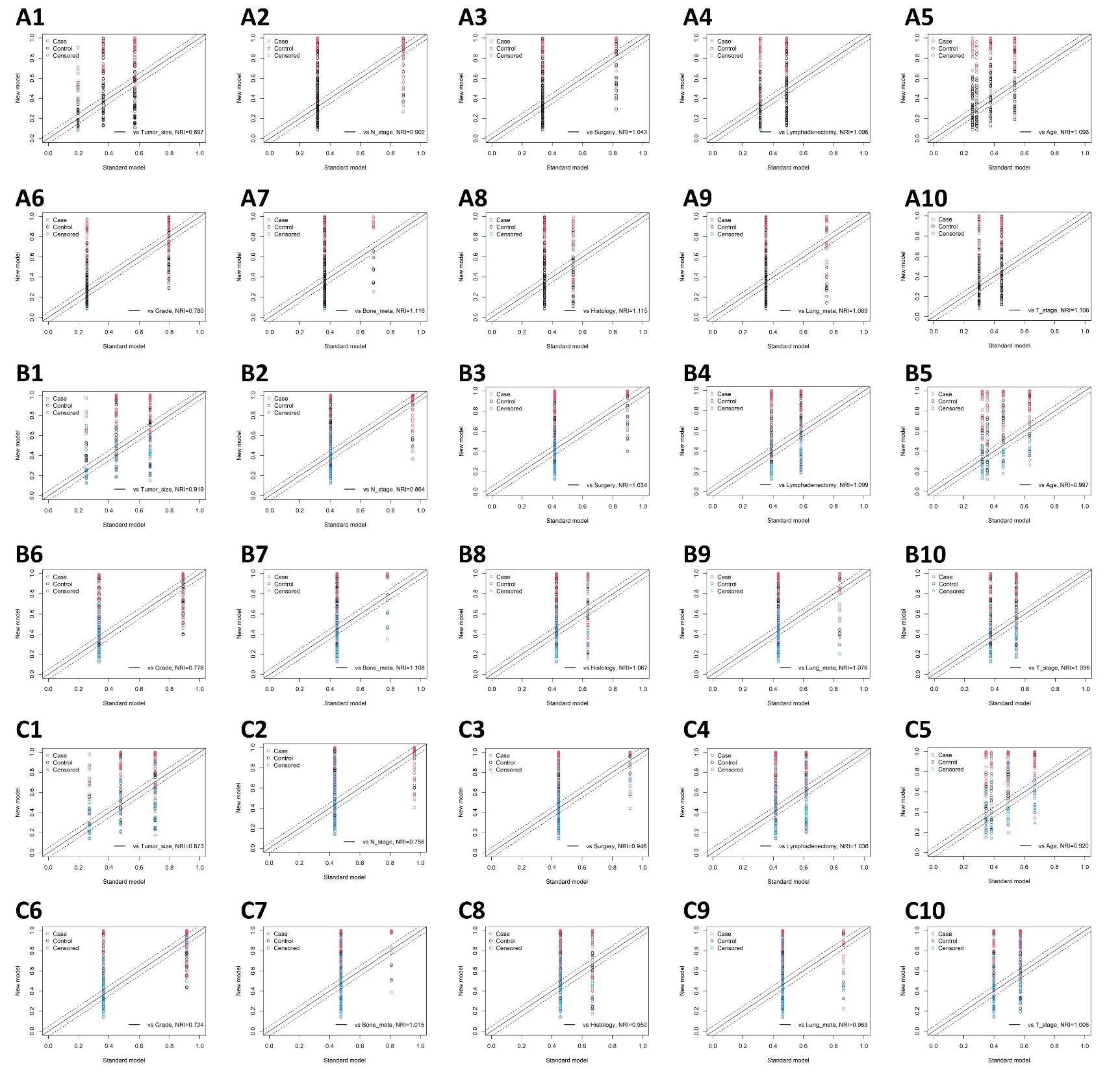


Supplementary Figure 3: Comparison of CSS nomogram with NRI for all independent factors for 3 years (A1-10), 5 years (B1-10) and 7 years (C1-10) in the training sets.


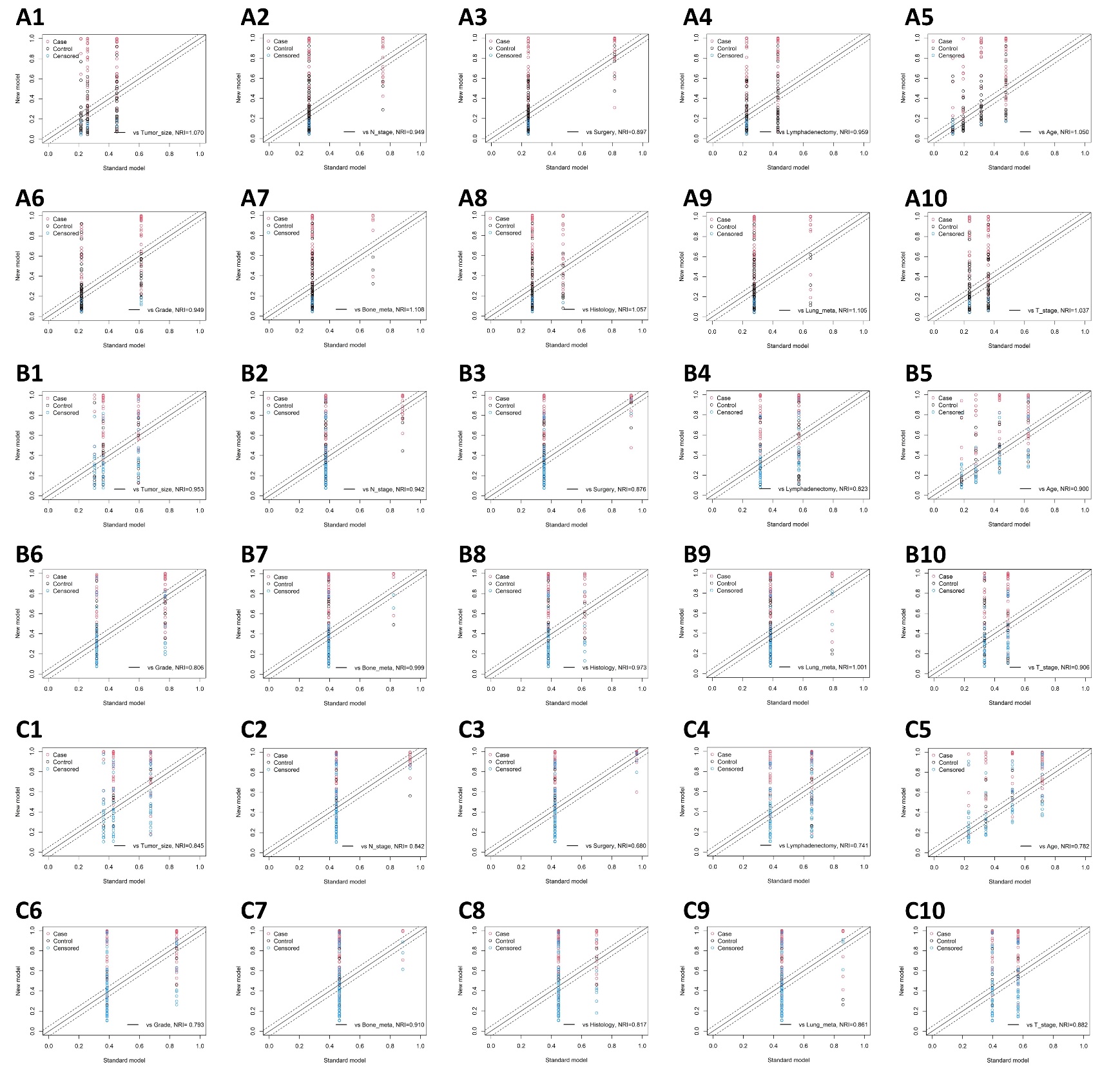


Supplementary Figure 4: Comparison of CSS nomogram with NRI for all independent factors for 3 years (A1-10), 5 years (B1-10) and 7 years (C1-10) in the validation sets.
